# Impact of Rotavirus Vaccine Introduction and Postintroduction Etiology of Diarrhea Requiring Hospital Admission in Haydom, Tanzania, a Rural African Setting

**DOI:** 10.1093/cid/cix494

**Published:** 2017-05-29

**Authors:** James A. Platts-Mills, Caroline Amour, Jean Gratz, Rosemary Nshama, Thomas Walongo, Buliga Mujaga, Athanasia Maro, Timothy L McMurry, Jie Liu, Estomih Mduma, Eric R Houpt

**Affiliations:** 1 Division of Infectious Diseases and International Health, University of Virginia, Charlottesville;; 2 Haydom Global Health Research Centre, Haydom Lutheran Hospital, and; 3 Kilimanjaro Clinical Research Institute, Moshi, Tanzania; and; 4 Department of Public Health Sciences, University of Virginia, Charlottesville

**Keywords:** rotavirus vaccine, effectiveness, diarrhea, children, PCR

## Abstract

**Background:**

No data are available on the etiology of diarrhea requiring hospitalization after rotavirus vaccine introduction in Africa. The monovalent rotavirus vaccine was introduced in Tanzania on 1 January 2013. We performed a vaccine impact and effectiveness study as well as a quantitative polymerase chain reaction (qPCR)–based etiology study at a rural Tanzanian hospital.

**Methods:**

We obtained data on admissions among children <5 years to Haydom Lutheran Hospital between 1 January 2010 and 31 December 2015 and estimated the impact of vaccine introduction on all-cause diarrhea admissions. We then performed a vaccine effectiveness study using the test-negative design. Finally, we tested diarrheal specimens during 2015 by qPCR for a broad range of enteropathogens and calculated pathogen-specific attributable fractions (AFs).

**Results:**

Vaccine introduction was associated with a 44.9% (95% confidence interval [CI], 17.6%–97.4%) reduction in diarrhea admissions in 2015, as well as delay of the rotavirus season. The effectiveness of 2 doses of vaccine was 74.8% (95% CI, –8.2% to 94.1%) using an enzyme immunoassay–based case definition and 85.1% (95% CI, 26.5%–97.0%) using a qPCR-based case definition. Among 146 children enrolled in 2015, rotavirus remained the leading etiology of diarrhea requiring hospitalization (AF, 25.8% [95% CI, 24.4%–26.7%]), followed by heat-stable enterotoxin-producing *Escherichia coli* (AF, 18.4% [95% CI, 12.9%–21.9%]), *Shigella*/enteroinvasive *E. coli* (AF, 14.5% [95% CI, 10.2%–22.8%]), and *Cryptosporidium* (AF, 7.9% [95% CI, 6.2%–9.3%]).

**Conclusions:**

Despite the clear impact of vaccine introduction in this setting, rotavirus remained the leading etiology of diarrhea requiring hospitalization. Further efforts to maximize vaccine coverage and improve vaccine performance in these settings are warranted.

## Summary

We describe the substantial impact of rotavirus vaccine introduction on all-cause and rotavirus diarrhea admissions to a rural Tanzanian referral hospital. Despite this impact, rotavirus remained the leading etiology of diarrhea requiring hospitalization in the third year after vaccine introduction.

Rotavirus remains the leading etiology of severe and fatal diarrhea worldwide, responsible for approximately 40% of diarrheal deaths [1]. The ongoing worldwide introduction of rotavirus vaccines is expected to have a substantial impact on the burden of rotavirus diarrhea [2]. Despite clinical trials and real-world effectiveness studies demonstrating reduced performance of these vaccines in sub-Saharan Africa [3–8], a profound impact of vaccine introduction has been observed, with the majority of the impact seen by the third year after introduction [9–12].

Because most prior etiologic studies have used a diverse group of diagnostic modalities, the advent of quantitative polymerase chain reaction (qPCR) diagnostics for enteropathogens has helped reveal an unbiased picture of the etiology of diarrhea in children in low-resource settings [13–14]. In particular, they have increased burden estimates for some pathogens, including *Shigella*, heat-stable enterotoxin-producing *Escherichia coli* (ST-ETEC), and adenovirus 40/41, but not others, including rotavirus and *Cryptosporidium*.

The relative importance of specific enteropathogens after rotavirus vaccine has been introduced is not known. We performed a study of the impact and effectiveness of vaccine introduction on all-cause and rotavirus diarrhea admissions to a rural African hospital, as well as a nested etiologic study using qPCR.

## METHODS

### Study Setting

The study was performed at Haydom Lutheran Hospital (HLH), a 450-bed referral hospital situated in a rural area in northern Tanzania, with a catchment area of approximately 2 million people [15]. The national immunization program is administered to this population through reproductive child health system clinics coordinated by HLH, which reach approximately 8000 new children per year [16]. The monovalent rotavirus vaccine was introduced to the national immunization program on 1 January 2013 for administration at 6 and 10 weeks of age.

### Study Design

To examine the impact of vaccine introduction on diarrhea admissions to HLH, we obtained data on all admissions for children <5 years of age to the pediatric ward from 1 January 2010 to 31 December 2015 from hospital discharge logs, including the date of admission, the child’s age, and the discharge diagnosis. A discharge diagnosis that included “gastroenteritis,” “diarrhea,” “dysentery,” or spelling variants thereof was coded as an admission for diarrhea. In addition, we performed a case-control vaccine effectiveness study, using the test-negative design, in which children with rotavirus diarrhea are included as cases and children with nonrotavirus diarrhea as controls [17]. Children who were admitted to the pediatric ward with diarrhea were screened for eligibility, and children who had at least 3 watery stools or 2 episodes of vomiting in a 24-hour period since the illness began, had a total duration of symptoms of <14 days, and were age-eligible to have received at least 1 dose of vaccine (ie, born on or after 1 October 2012 and at least 2 months of age) were approached for enrollment. Written consent was obtained from a parent or guardian. Demographic and clinical data were obtained from the parent or guardian by study personnel. Vaccine history was recorded directly from the child’s vaccine card. If the vaccine card was not available, and the parent or guardian reported that the child had never been vaccinated, the child was classified as having received no doses of vaccine. All other children without a vaccine card were excluded. A vaccine dose was counted if the admission occurred at least 14 days after administration. Ethical approval was obtained from the National Institute for Medical Research in Tanzania and the Institutional Review Board of the University of Virginia.

### Stool Testing and Case Detection

Collection of a stool was attempted within 48 hours of enrollment from all children, and all stool testing was performed on site in Haydom. Stool samples were tested for rotavirus by enzyme immunoassay (EIA) using the Oxoid ProSpecT Rotavirus test (Oxoid, Cambridge, United Kingdom) as well as by qPCR. For qPCR testing, nucleic acid was extracted with the QIAamp Fast DNA Stool mini kit (Qiagen, Hilden, Germany) using a modified protocol that included bead beating [18]. Two external controls, MS2 bacteriophage and phocine herpesvirus (PhHV), were added to samples during nucleic acid extraction to monitor extraction and amplification efficiencies. Extraction blanks and no-template controls were included to monitor for contamination. Testing was performed using a custom TaqMan Array Card, which provided simultaneous qPCR for a broad range of enteropathogens [19]. The primary enteropathogen assays on the card were identical to those used previously in the qPCR reanalysis of the Global Enteric Multicenter Study (GEMS) [13]. Additionally, qPCR typing was done for rotavirus G (1, 2, 3, 4, 8, 9, 10, and 12) and P (4, 6, 8, 9, 10, and 11) types and *Shigella* subtyping (*Shigella flexneri* type 6, non–type 6 *S. flexneri, Shigella sonnei*) using assays described previously [13, 20], as well as for enterotoxigenic *E. coli* colonization factors (CFA/1 and CS 1, 2, 3, 4, 5, 6, 7, 8, 12, 14, 17/19, and 18) using assays adapted from multiplex PCR panels [21, 22].

For the primary vaccine effectiveness analysis, cases were defined as children with diarrhea in whom rotavirus was detected by EIA, and the remainder of enrolled subjects were controls. For the secondary analysis, cases were defined as children with diarrhea in whom rotavirus was detected by qPCR with a quantification cycle (Cq) <32.6, a cutoff derived from the GEMS study to identify a highly diarrhea-associated quantity of rotavirus [13]. Highly diarrhea-associated cutoffs were also used to identify ST-ETEC and *Shigella*/enteroinvasive *E. coli* (EIEC) diarrhea episodes for describing the subtyping results, as well as to identify clinically significant coinfections in rotavirus episodes.

### Data Analysis

To determine the impact of vaccine introduction on all-cause diarrheal admissions to HLH, we performed an interrupted time-series analysis. We fit a quasi-Poisson model, to account for overdispersion, with an outcome of diarrhea admissions to HLH for each month; predictors included an exogenous trend, an intervention effect modeled as linearly increasing from 1 January 2013 to full effect on 31 December 2015, seasonality modeled via a second-order Fourier series by including the periodic terms $\text{sin}\left(\frac{2m\pi }{12}\right)$, $\text{cos}\left(\frac{2m\pi }{12}\right)$, $\text{sin}\left(\frac{4m\pi }{12}\right)$, and $\text{cos}\left(\frac{4m\pi }{12}\right)$, where *m* is the month of the year [23], and an interaction between the intervention effect and seasonality to account for observed shifting in seasonality post–vaccine introduction. We then calculated the reduction in diarrhea admissions in 2015 as the quotient of the model-predicted number of admissions and the predicted number of admissions with the intervention effect set to null. Confidence intervals were calculated with an autoregressive sieve-based residual bootstrap to account for remaining serial correlation in the data [24]. To estimate the impact of vaccine introduction on the seasonality of diarrhea admissions, we fit Poisson models for both the 3 years prior to and after vaccine introduction, with the number of admissions as the outcome and calendar month as the sole predictor. To calculate vaccine effectiveness, we fit an unconditional logistic regression model, with sex, age in months, year of admission, and season using the Fourier series described above included in the model a priori. Other covariates were retained in the model if they changed vaccine effectiveness estimates by >5%. Vaccine effectiveness was calculated as (1 – adjusted odds ratio) × 100%.

To estimate pathogen-specific burdens of diarrhea, we calculated an adjusted attributable fraction (AF) for each pathogen. First, we used models from the qPCR reanalysis of the GEMS study to derive quantity-specific odds ratios [13]. Specifically, using qPCR data from 5304 cases of moderate-to-severe acute diarrhea and age-, sex-, and village-matched controls, we fit a multivariable conditional logistic regression model to describe the association between pathogen quantity and diarrhea while adjusting for the presence of other pathogens. We then calculated AFs by summing pathogen attributions across each of *j* cases, that is, $\sum_{1}^{j}{\text{AF}}_{i}$, where ${\text{AF}}_{i}=1/\;j\times (1–1/{\text{OR}}_{i})$, and *OR*_*i*_ is the quantity-specific odds ratio derived from the regression model. To estimate the variance for the model-based attribution, the odds ratios were estimated 1000 times using random perturbations of the model coefficients in accordance with their sampling variance-covariance; these coefficients were drawn equally from each of the 7 GEMS sites. The 95% confidence intervals (CIs) were derived from the 2.5th and 97.5th quantiles of the AF distribution, and the point estimate of the AF was calculated using the original model coefficients.

## RESULTS

**Figure 1.**
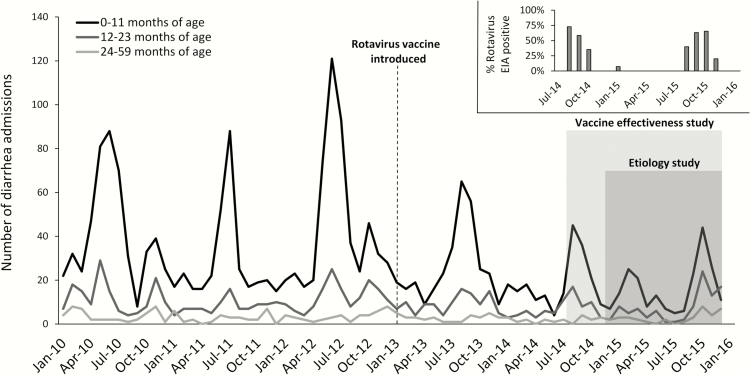
Monthly diarrhea admissions to Haydom Lutheran Hospital, January 2010–December 2015. Rotavirus vaccine was added to the national immunization program on 1 January 2013. The vaccine effectiveness study was performed from August 2014 to December 2015. Diarrhea etiology was assessed by quantitative polymerase chain reaction testing of all isolates from 2015. Inset (top right) shows the proportion of enrolled subjects who were positive for rotavirus by enzyme immunoassay (EIA) for each month.

**Figure 2.**
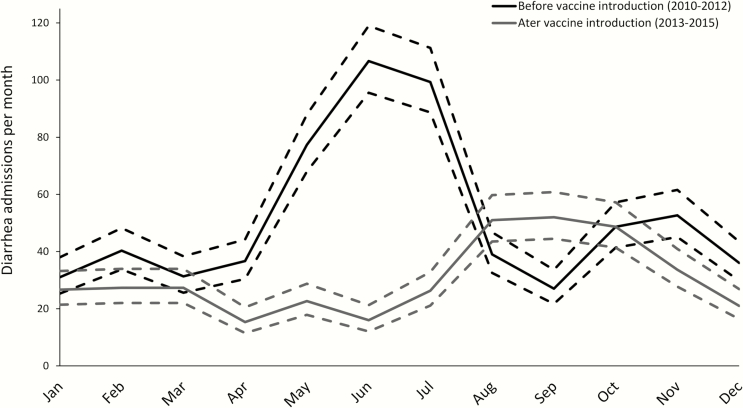
Monthly incidence of hospital admissions for diarrhea during 2010–2012, prior to vaccine introduction, and 2013–2015, after vaccine introduction. Incidence (solid lines) and 95% confidence intervals (dotted lines) were derived from Poisson regression models.

We recorded discharge diagnoses from 11258 hospital admissions of children <5 years of age from 1 January 2010 to 31 December 2015, of which 2971 (26.4%) were for diarrhea. Diarrhea admissions showed a striking seasonal pattern, with a clear reduction after vaccine introduction (Figure 1). Testing during the seasonal peaks in 2014 and 2015 for all children enrolled in the vaccine effectiveness study confirmed that rotavirus was the primary etiology of the seasonal peaks (Figure 1 inset). As expected, both the seasonality and the reduction after vaccine introduction were most striking in younger children. Additionally, a progressive delay in the rotavirus season was observed after vaccine introduction, from approximately May–July in 2010–2012 to August–October in 2013–2015 (Figure 2). Seasonal diarrhea appeared to be more pronounced in older children in 2015 than in previous years. Using an interrupted time-series analysis, in the third year after vaccine introduction, the estimated reduction in all-cause diarrhea cases in comparison to the predicted number of all-cause diarrhea cases based on prevaccine introduction trends was 44.9% (95% CI, 17.6%–97.4%).

**Figure 3.**
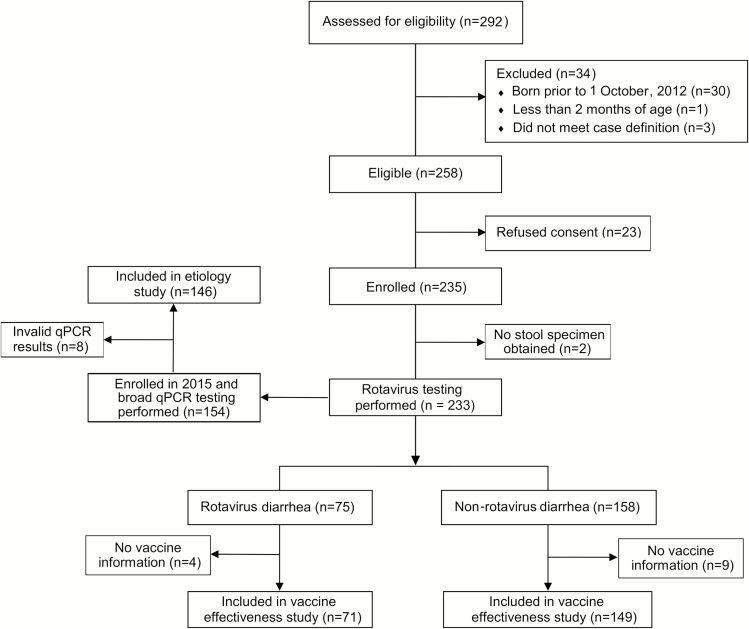
Flowchart for vaccine effectiveness and diarrhea etiology study. Rotavirus diarrhea was defined by for the primary analysis as detection of rotavirus by enzyme immunoassay and confirmed by quantitative polymerase chain reaction (qPCR).

We performed a vaccine effectiveness study from 19 August 2014 to 12 December 2015. Two hundred twenty children fulfilled the enrollment criteria and had both a stool sample tested for rotavirus and a vaccination history, of whom 71 (32.3%) met the primary rotavirus case definition (Figure 3). Possession of a mobile phone was more common among rotavirus cases, but other demographic characteristics were similar between cases and controls (Table 1). The median age of administration of the first and second dose of rotavirus vaccine was 10.6 (interquartile range [IQR], 8.3–12.7) weeks and 16.6 (IQR, 13.3–20.4) weeks, respectively. Vaccine coverage with 2 doses in controls was 121 of 149 (81.2%). Of the 71 rotavirus diarrhea cases enrolled during the effectiveness study, 62 were typed by PCR as G1P8, 8 as G1 with an undetermined P type, and a single case as G3P6. The effectiveness of 2 doses of the monovalent rotavirus vaccine was 74.8% (95% CI, –8.2 to 94.1%) using the primary EIA -based case definition and 85.1% (95% CI, 26.5%–97.0%) using the secondary qPCR-based case definition (Table 2).

**Table 1.** Demographic Characteristics of Rotavirus Diarrheal Cases and Nonrotavirus Diarrheal Controls

Characteristic	Rotavirus Diarrhea (n = 71)	Nonrotavirus Diarrhea (n = 149)	*P* Value
Age, mo^a^	9 (3–27)	10 (2–30)	.335
Male sex	42 (59.2)	78 (52.3)	.422
Prior hospital admission for diarrhea	22 (31.0)	37 (24.8)	.423
Home has a separate room for kitchen	40 (56.3)	88 (59.1)	.813
No. of bedrooms in home^a^	2 (1–4)	2 (1–4)	.278
No. of people sleeping in home^a^	7 (1–20)	6 (4–21)	.368
No. of children sleeping in home^a^	4 (1–8)	3 (1–13)	.459
Family owns a mattress	51 (71.8)	98 (65.8)	.457
Family owns a mobile phone	70 (98.6)	130 (87.2)	.013
Family owns a radio	38 (53.5)	79 (53.0)	1.000
From the Iraqw tribe	37 (52.1)	95 (63.8)	.133
Maternal age^a^	27 (16–47)	26 (17–41)	.919
Mother completed secondary school	50 (70.4)	87 (58.4)	.116
Protected drinking water source	27 (38.0)	70 (47.0)	.269
Child still breastfeeding	63 (88.7)	119 (79.9)	.151

Data are presented as No. (%) unless otherwise indicated. The Wilcoxon rank-sum test was used for all continuous variables and the χ^2^ test was used for all dichotomous variables. Maternal age was missing for 4 subjects.

^a^Median (interquartile range).

**Table 2.** Effectiveness of 2 Doses of Rotavirus Vaccine Against Admission to Haydom Lutheran Hospital With Rotavirus Diarrhea Among Age-Eligible Children, 19 August 2014 to 12 December 2015

Case Definition	Rotavirus Diarrhea (n = 71)	Nonrotavirus Diarrhea (n = 149)	Adjusted VE, % (95% CI)^b^
EIA positive			
0 doses	9 (12.7)	10 (6.7)	
2 doses	57 (80.3)	121 (81.2)	74.8 (–8.2 to 94.1)
Highly diarrhea-associated rotavirus detection by qPCR^a^			
0 doses	10 (13.3)	9 (6.2)	
2 doses	59 (78.7)	119 (82.1)	85.1 (26.5–97.0)

Data are presented as No. (%) unless otherwise indicated.

Abbreviations: CI, confidence interval; EIA, enzyme immunoassay; qPCR, quantitative polymerase chain reaction; VE, vaccine effectiveness.

^a^Highly diarrhea-associated rotavirus detection was defined as qPCR detection with quantification cycle <32.6. For this case definition, 75 of 220 (34.1%) were classified as cases.

^b^Adjusted for age, sex, year of admission, and seasonality.

**Figure 4.**
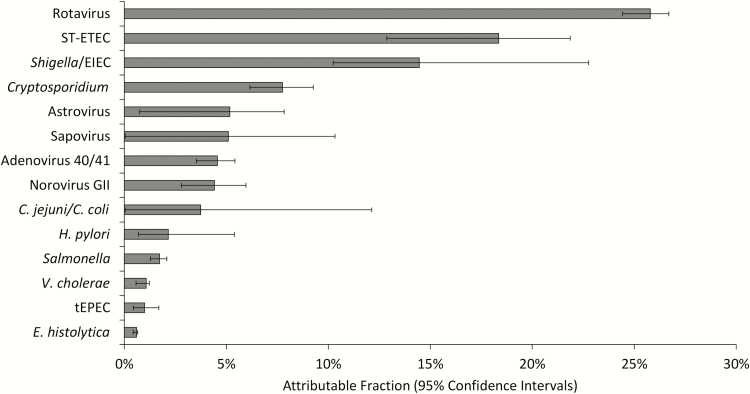
Etiology of diarrhea requiring hospitalization in 2015 by quantitative polymerase chain reaction (qPCR) (n = 146). Attributable fraction estimates were derived from qPCR detection of each pathogen combined with odds ratios derived from the Global Enteric Multicenter Study (GEMS). Abbreviations: *C. coli*, *Campylobacter coli*; *C. jejuni*, *Campylobacter jejuni*; *E. histolytica*, *Entamoeba histolytica*; EIEC, enteroinvasive *Escherichia coli*; *H. pylori*, *Helicobacter pylori*; ST-ETEC, heat-stable enterotoxin-producing *Escherichia coli*; tEPEC, typical enteropathogenic *Escherichia coli*; *V. cholerae*, *Vibrio cholerae*.

For the nested etiology study, 154 subjects were enrolled in 2015, the third year after vaccine introduction. Stool specimens from each subject were tested by qPCR, and 146 (94.8%) had valid qPCR results for analysis. The median age was 10 (IQR 8–15) months, with 140 of 146 (95.9%) <2 years of age. A vaccination history was available for 135, of whom 113 (83.7%) had received 2 doses, 15 (11.1%) had received 1 dose, and 7 (5.2%) were unvaccinated. Using the GEMS models to attribute pathogen detection as the etiology of each diarrhea episode, rotavirus remained the leading etiology of diarrhea requiring hospitalization (AF, 25.8% [95% CI, 24.4%–26.7%]), followed by ST-ETEC (AF, 18.4% [95% CI, 12.9%–21.9%]), *Shigella*/EIEC (AF, 14.5% [95% CI, 10.2%–22.8%]), and *Cryptosporidium* (AF, 7.9% [95% CI, 6.2%–9.3%]) (Figure 4). More than two-thirds (69.2%) of all diarrhea attribution was to these 4 pathogens. The rotavirus AF was similar for the 90 of 146 (61.6%) children <12 months of age (AF, 26.3% [95% CI, 25.0%–27.3%]) and the 56 (38.4%) aged ≥1 year (AF, 24.8% [95% CI, 23.3%–25.8%]). In 2015, 42 of 146 episodes (28.8%) had rotavirus detected at a highly diarrhea-associated quantity by qPCR, of which 38 of 42 (90.5%) were positive by EIA and 32 of 42 (76.2%) had no other pathogens detected at a highly diarrhea-associated quantity. In the remaining 10 episodes, a single highly diarrhea-associated copathogen was identified in each, namely *Cryptosporidium* [1], *Helicobacter pylori* [1], ST-ETEC [4], *Shigella*/EIEC [2], and *Vibrio cholerae* [2]. Only 2 of 42 (4.8%) were admitted within 1 month of their most recent rotavirus vaccine dose.

**Figure 5.**
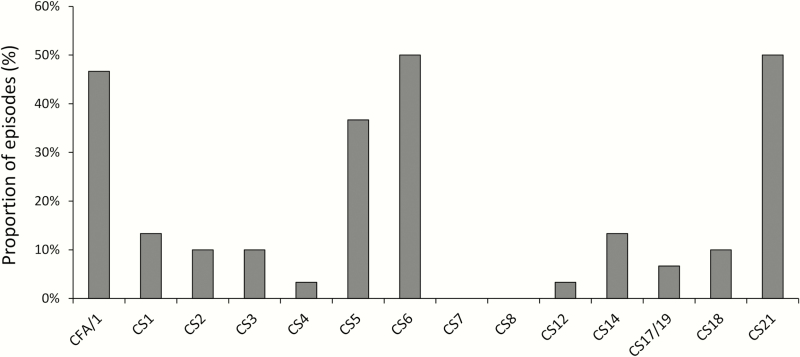
Heat-stable enterotoxin-producing *Escherichia coli* colonization factor profile for all highly-diarrhea associated detections in 2015 by quantitative polymerase chain reaction. Abbreviations: CFA, colonization factor antigen; CS, coli surface antigen.

To perform subtyping for ST-ETEC and *Shigella* to help inform vaccine development, we used quantitative cutoffs derived from the GEMS study to identify ST-ETEC and *Shigella*-associated diarrhea [13]. ST-ETEC was detected at a quantity greater than the cutoff for 30 of 146 diarrhea episodes (20.5%), and the predominant colonization factors were CFA/1, CS5, CS6, and CS21 (Figure 5). CS21 was detected in 15 of 30 (50.0%) ST-ETEC-associated episodes. Of those, CFA/1 was detected in 12 of 15 (80.0%). One or more of CFA/I, CS 3, CS 5, and CS6 was present in 24 of 30 (80.0%). *Shigella* was detected at a quantity greater than the cutoff for 20 of 146 diarrhea episodes (13.7%), of which 8 (40.0%) were classified as *S. flexneri* (non–type 6), 4 (20.0%) were classified as *S. sonnei*, and 8 (40.0%) could not be subtyped by qPCR.

## DISCUSSION

In a rural Tanzania referral hospital, despite a clear and substantial impact of vaccine introduction on all-cause diarrhea admissions and high vaccine effectiveness, rotavirus remained the leading etiology of diarrhea in the third year after vaccine introduction. This highlights the critical importance of ongoing work to optimize the performance of rotavirus vaccination in these settings. In addition to efforts to maximize vaccine coverage to increase herd immunity, additional research on the design, timing, and delivery of rotavirus vaccination [25–29], as well as identification of modifiable factors associated with poor vaccine immunogenicity [30–31], is warranted. A wide range of potential strategies have been identified, including zinc supplementation, withholding of breastfeeding, additional doses including neonatal dosing, avoiding coadministration with oral polio vaccine, and parenteral vaccination [29].

The attenuation and delay in seasonal diarrhea, as well as the reduction in all-cause diarrhea admissions, are consistent with a substantial impact of vaccine introduction. The reduction in all-cause diarrhea admissions was similar to that described in South Africa and Rwanda [10, 12] and suggests that if rotavirus caused 25% of diarrhea requiring admission after vaccine introduction, it was likely responsible for 50%–60% prior to introduction. The striking delay in the rotavirus season is consistent with data from the United States [32], and is predicted by modeling studies [33], but has not been observed in other studies of vaccine introduction in Africa. This is suggestive of strong herd immunity in this rural, isolated population. However, in rotavirus-negative controls from August 2014 to December 2015, 81.2% had received 2 vaccine doses. This is lower than the World Health Organization/United Nations Children’s Fund estimates for country-wide coverage with a complete course of rotavirus vaccine in Tanzania of 85% in 2014, 97% in 2015, and 98% in 2016 [34]. It is possible that coverage in the most rural areas is lower, which has implications for the nationwide impact of vaccine introduction [35]. Relaxation of the age restrictions for receiving rotavirus vaccine could also lead to improved coverage [36–37].

The vaccine effectiveness point estimates were high in this study. Other studies from East Africa have ranged from 57% to 75% [6, 8, 37]. While the precision in all of these studies is relatively low, there are several potential reasons for the high point estimates noted here. First, vaccine delivery was later than recommended by schedule. A clinical trial of the monovalent rotavirus vaccine with a delayed dosing strategy (ie, 10 and 17 weeks) demonstrated higher efficacy than was seen in comparable trials in these settings [25], while the addition of a third, later dose was associated with increased immunogenicity in comparison to a reference 2-dose schedule [39]. Declining interference from maternal immunity may explain these observations [30]. Second, the vast majority of the circulating rotavirus in this population was of the vaccine-homotypic G1P8 strain. The only evaluation of strain-specific effectiveness in Africa to date suggested a higher effectiveness against homotypic strains [6]. Vaccine effectiveness was similar between EIA- and qPCR-based case definitions, suggesting that the EIA is sufficiently sensitive to detect most clinically significant rotavirus infections. The use of qPCR for this pathogen has not significantly changed population-level burden estimates [13].

In this study, *Shigella*, ST-ETEC, and *Cryptosporidium* were also important etiologies of diarrhea requiring hospitalization and are potential targets for further reduction in diarrhea burden with pathogen-specific interventions. The ST-ETEC colonization factor profile in this study is consistent with prior data suggesting that CFA/1, CS3, CS5, and CS6 account for a majority of all CF-positive clinical ETEC isolates and that CS21 is emerging as a colonization factor of global importance [40–42]. Because CS21 and CFA/1 were frequently codetected, current ETEC vaccine candidates that target CFA/I, CS3, CS4, CS5, and CS6 would still be predicted to cover 80% of the diarrhea-associated ETEC in this study. The majority of *Shigella*-associated diarrhea episodes were subtyped as *S. flexneri* or *S. sonnei*, which are the primary targets of *Shigella* vaccine development [43]. These data would suggest that a combination vaccine against ETEC and *Shigella* would be of considerable value in the post–rotavirus vaccine era [44]. The finding of a high burden of *Cryptosporidium* diarrhea is consistent with findings from GEMS that this is a pathogen of particular importance in African infants [13].

This study had several limitations. Because the etiologic study was nested in a vaccine effectiveness study and was limited to age-eligible children, infants predominated, an age group for which rotavirus has been observed to be more dominant [13, 45]. Therefore our attribution of diarrhea to rotavirus may be inflated in comparison to all hospitalized children <5 years of age with diarrhea. However, rotavirus burden estimates were similar for infants and older children, and the age composition of the seasonal peak in diarrhea admissions appeared to shift toward older children in 2015, which has been observed elsewhere [46]. Additionally, while it is possible that vaccine shedding after vaccine introduction might lead to the false attribution of diarrhea etiology to rotavirus, the vast majority of the children enrolled in the etiologic study were remote from vaccine administration. Furthermore, rotavirus remained the leading etiology of diarrhea even after exclusion of all episodes in which diarrhea-associated coinfections were detected. Because subclinical carriage of these pathogens is common in these settings, to determine pathogen-specific attribution, we extrapolated from models developed from the GEMS study, which included 7 sites from Africa and Asia, and these models may not be generalizable. However, our estimates account for the variance in pathogen carriage across all of these sites, and our study setting has been shown to be similar in terms of pathogen exposure and carriage to other sites in Africa and Asia [45]. For some pathogens, burden estimates were imprecise, in particular *Campylobacter,* sapovirus, and norovirus GII. This is in part due to the sample size, but also due to the frequent asymptomatic carriage of these pathogens, which hampers precision using the AF approach. However, this methodology remains strongly preferable to the alternative of ascribing etiology solely based on the cross-sectional detection of pathogens in diarrhea episodes [13, 45].

This study adds to the growing evidence of an important and sizeable impact of rotavirus vaccine introduction in Africa and supports the ongoing effort for global introduction. Efforts to ensure optimal vaccine coverage, even in the most rural areas, are critical. However, despite relatively high coverage and high effectiveness, we find that rotavirus remained the leading etiology of diarrhea requiring hospitalization in this rural African setting, and further work to improve vaccine performance in these settings is needed. ST-ETEC, *Shigella*, and *Cryptosporidium* appear to be important pathogens to target to further reduce the burden of diarrheal disease in Africa.

## Notes

***Acknowledgments*.** We thank the families and children who participated in this study and the administration and personnel at Haydom Lutheran Hospital.

***Financial support.*** This study was supported by the Bill & Melinda Gates Foundation (grant number OPP1019093 to E. R. H.). J. P. M. was supported by the National Institutes of Health (grant number K23 AI114888) and an *American Society of Tropical Medicine and Hygiene*/Burroughs Welcome Fund Postdoctoral Fellowship in Tropical Infectious Diseases.

***Potential conflicts of interest*.** All authors: No reported conflicts of interest. All authors have submitted the ICMJE Form for Disclosure of Potential Conflicts of Interest. Conflicts that the editors consider relevant to the content of the manuscript have been disclosed.
